# 2025 Acknowledgment of JCM Reviewers

**DOI:** 10.1128/jcm.00341-26

**Published:** 2026-04-08

**Authors:** Romney M. Humphries

**Affiliations:** 1 Vanderbilt University Medical Center 12328, Nashville, Tennessee, USA

## EDITORIAL

The *Journal of Clinical Microbiology*’s high quality and timely publication of groundbreaking scientific research are critically dependent on the outstanding efforts of the editorial board members (https://journals.asm.org/journal/jcm/board-editors) who generously contribute their time, wisdom, and talents to our journal. The editors of JCM also acknowledge the *ad hoc* referees (listed below) who have contributed reviews in the past year, and we express our deep appreciation for their contributions to maintaining the journal’s scientific rigor, relevance of research, and high clinical impact.

Davide A. Abate

April Abbott

La'Tonzia L. Adams

Ritesh Agarwal

Olusola Anuoluwapo Akanbi

Saleem Khteer Al-hadraawy

David C. Alexander

Thomas S. Alexander

Ibne Karim M. Ali

John Alverdy

Melis N. Anahtar

Sönke Andres

Andrea Angheben

Alicia Aranaz

Derek T. Armstrong

Paul M. Arnaboldi

Chris Attaway

Htin Lin Aung

Paul Gisbert Auwaerter

Luiza Aymee

Hamid Badali

Ritu Banerjee

Mike Barer

Allen C. Bateman

Marcel A. Behr

Ciresthel Bello Rios

Gabriele Bianco

Martina Bielaszewska

Michael Biggel

Fabiana Bigi

Patrick J. Blackall

April Bobenchik

Andrew M. Borman

Rachel E. Bosserman

David Boulware

John Branda

Tobias Broger

Barbara A. Brown-Elliott

Cameron A. Brown

Daniel R. Brown

Garrett Brown

Andrew Bryan

Emily Bryan

Kendall Bryant

Patrick W. Bryant

Blake Wade Buchan

Frederick S. Buckner

Bryce M. Buddle

Tina I. Bui

Dora Buonfrate

Carey-Ann D. Burnham

Karen Bush

Angela M. Caliendo

Emmanuelle Cambau

Shelley Campeau

Victoria L. Campodónico

Gerard A. Cangelosi

Matthew Cappiello

Giuseppina Capra

Gianluigi Cardinali

Heather A. Carleton

Karen C. Carroll

Sandrine Castelain

Robin R. Chamberland

Ka Pang Chan

Kwok-Hung Chan

Day-Yu Chao

Angella Charnot-Katsikas

Charlotte Charpentier

Sudha Chaturvedi

Derrick J. Chen

Ty Chiaro

Charles Y. Chiu

Daniela Maria Cirillo

Ousmane H. Cissé

Cornelius J. Clancy

Shawn Clark

Shawn T. Clark

Renee Codsi

Tom Coenye

Daniel Cohen

Joseph M. Colacino

Stephen D. Cole

Jessica Colon-Franco

Emilyn Costa Conceicao

Caleb Cornaby

Matheus Costa

Cristina Costales

Natacha Couto

Lindsey B. Crawford

Hannah M. Creager

Israel Cruz

Gregory Curler

Antonio Curtoni

Alexandre J. Da Silva

Ana Paula Da Silva

Bouke C. de Jong

Boudewijn DeJonge

Keith M. Derbyshire

Marie Desnos-Ollivier

Sridevi Devaraj

Courtney N. Dial

Dubraska V. Diaz-Campos

Karl Dichtl

Pedro Paulo Vissotto de Paiva Diniz

Samuel Dominguez

James J. Dunn

Paul H. Edelstein

Koray Ergunay

Erdal Erol

Ana V. Espinel-Ingroff

Joseph O. Falkinham

Miguel Fernández-Huerta

Javier Fernández

Mark A. Fisher

Ola Forslund

Dimitrios Frangoulidis

Thomas R. Fritsche

Stefan Fuchs

Hans-Peter Fuehrer

Jean-Pierre Gangneux

Yulong Gao

Miguel Angel Garcia Bereguiain

Waldo Luis García-Jiménez

Andrea Garolla

Goran Glodic

Richard V. Goering

David M. Goldfarb

Wenping Gong

Jose L. Gonzales

Eamonn Gormley

Erin Helen Graf

Dan Gregson

Thomas E. Grys

Jeannette Guarner

Damitha Gunathilake

Ankur Gupta-Wright

Ferry Hagen

Sven Halbedel

Sagarika Haldar

Anette M. Hammerum

Dongsheng Han

Xiao Han

Julia D. Hankins

Timm C. Harder

D. Jane Hata

Marie-Pierre Hayette

David Hillyard

Janet A. Hindler

Sabine Hofmann-Thiel

Diana Huang

Yosef Daniel Huberman

Kristina G. Hulten

Klaus-Peter Hunfeld

Ashraf S. Ibrahim

Evgeny A. Idelevich

Nazir Ismail

Naoto Ito

Michael R. Jacobs

Alexis Jaramillo Cartagena

Joseph Nicholas Jarvis

Byung Woo Jhun

Kyung-Wook Jo

J. Kristie Johnson

Nicholas Johnson

Stephen J. Jordan

Maria José Borrego

Justin E. Juskewitch

Sheila M. Keating

Ayesha Khan

Christine M. Khosropour

Sarah E. Kidd

Hyon-Suk Kim

James E. Kirby

Laura M. Koeth

Mikashmi Kohli

Kinga T. Kowalewska-Grochowska

Barry N. Kreiswirth

Peter Kuhnert

Ed J. Kuijper

Anil Kumar

Nakwon Kwak

Coralie L'Ollivier

Oliver Laeyendecker

Katrien Lagrou

Hanne Lamberink

Daryl M. Lamson

Marie L. Landry

Pascal Lapierre

Paige M. K. Larkin

Maureen Laroche

Peter Lasch

Thuy Le

Sixto M. Leal

Tessa E. LeCuyer

Sebastien Lhomme

Stuart Lichtenberg

Sabine Lichtenegger

Joshua A. Lieberman

Oliver Liesenfeld

Qingyun Liu

Ze-Hu Liu

Erley Ferlipe Lizarazo

Michael J. Loeffelholz

Kavita S. Lole

Oliver Lorenz

Rogier Louwen

Christopher Fong Jen Lowe

John Dustin Loy

Jacky Lu

Lungen Lu

Martin Ludlow

Ky-Phuong Luong

Yuan Lyu

Angela Ma

Ruihong Ma

Emily MacLean

Gundallahalli Bayyappa Manjunathareddy

Brendon Coenrad Mann

Antonella Marangoni

Michael Marks

Adriana R. Marques

Grace Marx

Ponpan Matangkasombut

Amy J. Mathers

Yasufumi Matsumura

Erika Matuschek

Alexander J. McAdam

Lesley McGee

Bradford S. McGwire

Michael T. McIntosh

Nicholas J. Mercuro

Folker Meyer

Hamed Mirjalali

Anisha Misra

Michel Monod

Jose Montoya

Rodrigo Morales

Eileen Moran

Muhammad G. Morshed

Beat Mueller

Mick N. Mulders

Thomas Müller

Erik Munson

David R. Murdoch

Shannon G. Murphy

Emmanuel Musisi

Kimberlee A. Musser

Munyaradzi Musvosvi

Denys Muzyka

Omary Mzava

Samia N. Naccache

Sandra Naegele

Moon H. Nahm

Kevin A. Nash

Tara E. Ness

Terry Fei Fan Ng

Denver T. Niles

Andrew P. Norgan

Priya Nori

Anne-cécile Normand

Laura Olbrich

Francisco Olea-Popelka

Randall J. Olsen

Kenji Ota

Caitlin Otto

Gudrun Overesch

Gustavo F. Palacios

Tara N. Palmore

Subrat Kumar Panda

Marcus Panning

Costas C. Papagiannitsis

John R. Papp

Elena Pariani

Bijal A. Parikh

Kun Taek Park

Alexander O. Pasternak

David Payne

Rosanna W. Peeling

Vera Lucia Pereira-Chioccola

Olivia Peuchant

Giulia Pezzoni

Dylan R. Pillai

Sylvie Pillet

Benjamin A. Pinsky

Corentin Poignon

Laurent Poirel

Nira R. Pollock

Mark Aaron Poritz

Bruno Pozzetto

Neeta Pradhan

Xiaotong Qiu

Flavio Queiroz-Telles

Yvonne Qvarnstrom

Thandavarayan Ramamurthy

Magnus Rasmussen

Timothy D. Read

Susan Realegeno

Carlos Reding

Rui-Wen Ren

Chantal B. E. M. Reusken

Stefan Riedel

Leen Rigouts

Florence Robert-Gangneux

Esther Robinson

David Rodriguez-Temporal

Timothy C. Rodwell

Robert Joseph Rolfe

Eduard Roos

John W. A. Rossen

Andrew N. Rycroft

Claude Sabeta

Raquel Sabino

David A. Sack

Max Salfinger

Stephen Salipante

Vittorio Sambri

Linoj Philip Samuel

Belkys Cecilia Sanchez

Maurizio Sanguinetti

Michael Joseph Satlin

Shingo Sato

Frieder Schaumburg

Mark R. Schleiss

Jonas Schmidt-Chanasit

John L. Schmitz

Anna Schotthoefer

Inna Sekirov

Amir Seyedmousavi

Nazly Shafagati

Salika M. Shakir

Sadia Shakoor

Shamanth Shankarnarayan

Rohini Sharma

Ismaila Shittu

Francesco Simonetti

Binit Kumar Singh

Durg Vijai Singh

Derek D. N. Smith

Sveinung Wergeland Sørbye

Ruan Spies

Kalpana Sriraman

Willy Ssengooba

Irene A. Stafford

Meghan Starolis

Kathleen A. Stellrecht

Milan S. Stosic

Franc Strle

Kaede V. Sullivan

Huiling Sun

Alexander Sundermann

Neeti Swarup

Jane E. Sykes

Wendy A. Szymczak

Sabira Tahseen

Chao Tang

Nicole J. Tarlton

Phillip Tarr

Marcus de Melo Teixeira

Devina Josephine Thiono

Matthew James Thoendel

Phyu M. Thwe

Robert J. Tibbetts

María Teresa Tórtola Fernández

Arthur H. Totten

Thao T. Truong

Sarah E. Turbett

Tam T. Van

Esther Vaugon

Paschalis Vergidis

Chaitenya Verma

David Veyer

Isabelle Villena

Jan Vinje

Benoit Visseaux

L. Lutz von Müller

Debra A. Wadford

Retno Wahyuningsih

David H. Walker

Thomas J. Walsh

David Wang

Hannah Wang

Mao-Shui Wang

Quanyi Wang

Shujie Wang

J. Scott Weese

L. F. Westblade

A. Clinton White Jr.

Philip Lewis White

Dana G. Wolf

Gwendolyn E. Wood

Christopher W. Woods

Shenghai Wu

Lihua Xiao

Lan Xu

Pablo Yagupsky

Shangxin Yang

Hyunah Yoon

Peixia Yu

Kwok-Yung Yuen

Man-Fung Yuen

Jinhua Zhang

Wei Zhang

